# Association of *VEGFA* and *IL1β* gene polymorphisms with preeclampsia in Sudanese women

**DOI:** 10.1002/mgg3.1119

**Published:** 2020-01-14

**Authors:** Hameed M. Hamid, Sana E. Abdalla, Mohamed Sidig, Ishag Adam, Hamdan Z. Hamdan

**Affiliations:** ^1^ Faculty of Medicine Al‐Neelain University Khartoum Sudan; ^2^ Al‐Neelain Institute for Medical Research (NIMR) Al‐Neelain University Khartoum Sudan; ^3^ Department of Obstetrics and Gynecology Unaizah College of Medicine Qassim University Unaizah Kingdom of Saudi Arabia

**Keywords:** gene polymorphisms, interleukin 1 beta, preeclampsia, Sudan, vascular endothelial growth factor A

## Abstract

**Background:**

Preeclampsia can lead to adverse maternal and perinatal outcomes. There are few studies on the genetic factors associated with preeclampsia in Africa in general and in Sudan in specific.

**Methods:**

A case–control study (60 women in each arm) was conducted at Saad Abualila Hospital in Khartoum, Sudan, from March to September 2018. The participants were genotyped for vascular endothelial growth factor A (*VEGFA*) rs3025039, interleukin 1 beta (*IL1β)* rs16944, and *IL1β* rs1143634 by performing polymerase chain reaction–restriction fragment length polymorphism analysis, and the results were confirmed by DNA sequencing.

**Results:**

There was no significant difference in the age, parity, body mass index, or other characteristics tested between the preeclampsia group and the control group (60 women in each arm). The rs3025039, rs16944, and rs1143634 genotypes were distributed in accordance with Hardy–Weinberg equilibrium (*p* > .05). For rs3025039, CT, CT+TT, and the T allele were risk factors for preeclampsia (odds ratio [OR] = 2.4; 95% CI [1.12–5.32]; *p* = .02; OR = 2.49 [1.17–25.27]; *p* = .01; OR = 2.05; 95% CI [1.10–3.83]; *p* = .02, respectively). Regarding rs16944, only the heterozygous genotype CT was associated with preeclampsia (OR = 2.55; 95% CI [1.15–5.56]; *p* = .01). Regarding rs1143634, CT, CT+TT, and the T allele were risk factors for preeclampsia (OR = 5.28; 95% CI [2.26–12.33]; *p* < .001; OR = 4.50; 95% CI [2.06–9.81]; *p* < .001; OR = 2.75; 95% CI [1.48–5.12]; *p* = .001, respectively).

**Conclusion:**

Polymorphisms in *IL1β* and *VEGFA* were associated with preeclampsia in this setting. Significant associations were observed between preeclampsia and rs3025039, rs16944, and rs1143634.

## Graphical abstract

Preeclampsia can lead to adverse maternal and perinatal outcomes. We investigate single nucleotide polymorphisms in vascular endothelial growth factor A (VEGFA) and interleukin‐1β. Significant association was observed between preeclampsia and rs3025039, rs16944, and rs1143634.

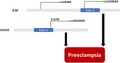

## 1 INTRODUCTION

Preeclampsia is a multisystem disorder defined as hypertension and significant proteinuria after the 20th week of gestation in a previously normotensive woman (American College of Obstetricians and Gynecologists, 2002). It is a major disease associated with pregnancy and occurs in approximately 3%–8% of all human pregnancies (Abalos, Cuesta, Grosso, Chou, & Say, 2013; Lo, Mission, & Caughey, 2013).

Although the exact pathophysiology/etiology of preeclampsia is not fully understood, preeclampsia is characterized by inadequate development of the early placenta, known as “poor placentation,” and reduced utero‐placental circulation capacity, which can lead to endothelial dysfunction and the initiation of preeclampsia (Redman, 1991; Redman & Sargent, 2005).

Various genetic variations are known to be associated with preeclampsia; however, the precise pathological mechanism and genetic basis of preeclampsia remain unknown (Lisowska, Pietrucha, & Sakowicz, 2018). Vascular endothelial growth factor A (*VEGFA*, OMIM: 192240) is essential for placental development and vascularization (Ferrara & Davis‐Smyth, 1997). VEGFA is the gene product of *VEGFA*, which is located on chromosome 6 at cytogenetic band 6p21.3 (Takahashi & Shibuya, 2005). The association of rs3025039 (NC_000006.12:g.43784799C>T) with preeclampsia and its possible role in the pathogenesis of preeclampsia have been previously investigated (Andraweera et al., 2013a; Chedraui et al., 2013). However, the results remain inconclusive and contradictory (Andraweera et al., 2013a; Shim et al., 2007).

High levels of interleukin‐1β (IL‐1β) are associated with preeclampsia (Mulla et al., 2011; Rinehart et al., 1999). IL‐1β triggers the expression of cytokines that induce the pro‐inflammatory cascade reaction that leads to release of many inflammatory cytokines (Visser, Beckmann, Knook, & Wallenburg, 2002). The gene encoding IL‐1β, *IL1β* (OMIM: 147720), is located at cytogenetic band 2q14 on chromosome 2 (“Gene: IL1B (ENSG00000125538) ‐ Summary ‐ Homo sapiens ‐ Ensembl genome browser 95,” n.d.). Results from studies investigating the association of rs16944 (NG_008851.1:g.4490T>C) with preeclampsia have been contradictory (Kang, Chen, Yu, Chang, & Chang, 2012; Nasr, El Azizy, Hassan, Salem, & Diaa, 2017; Wang et al., 2014). However, no study has shown an association between rs1143634 (NG_008851.1:g.8967C>T) and preeclampsia (Leme Galvão et al., 2016; Mohajertehran, Tavakkol Afshari, Rezaieyazdi, & Ghomian, 2012; Tavakkol Afshari et al., 2016).

Preeclampsia/eclampsia is a major health problem that is associated with high maternal and perinatal mortality in Sudan (Ali, Rayis, Abdallah, Abdullahi, & Adam, 2011; Ali, Okud, Khojali, & Adam, 2012). We recently reported a significant association between *F5* (NG_011806.1:g.41721G>A) and preeclampsia in Sudanese women (Ahmed, Adam, Elzaki, Awooda, & Hamdan, 2019). Moreover, we previously showed that VEFGA is expressed at significantly higher levels in the placentas of preeclamptic women compared with the placentas of healthy women (49.6% vs. 14.9%) (Ali, Salih, Elhassan, Mohmmed, & Adam, 2019). There are few published data regarding the association of *VEGFA* and *IL1β* polymorphisms with preeclampsia (Shim et al., 2007; Tavakkol Afshari et al., 2016; Wang et al., 2014), and there are no published data regarding the association of *VEGFA* and *IL1β* polymorphisms were preeclampsia in sub‐Saharan African populations, including in Sudan. Therefore, the aim of this study was to assess the association of single nucleotide polymorphism (SNP) rs3025039, rs16944, and rs1143634 with preeclampsia in Sudanese women.

## 2 MATERIALS AND METHODS

### 2.1 Ethical compliance

The study received ethical clearance from the Al‐Neelain University Ethics review board. All participants provide signed informed consent before enrollment.

### 2.2 Study subjects

A case–control study (60 women in each arm) was conducted at Saad Abualila Maternity Hospital in Khartoum, Sudan, from March to September 2018.

The preeclampsia group included pregnant women with a blood pressure ≥140/90 mmHg on two occasions, at least 6 hr apart, and proteinuria of ≥300 mg/24 hr. Preeclampsia subtypes were classified as mild to severe, with severe preeclampsia defined as having a blood pressure ≥160/110 mmHg on two occasions, at least 6 hr apart, proteinuria of ≥5 g/24 hr, and HELLP syndrome (hypertension, proteinuria and presence of hemolytic anemia, elevated liver enzymes, and low platelet count) (American College of Obstetricians and Gynecologists, 2002).

Healthy pregnant women without hypertension, proteinuria, diabetes, or any underlying disease were used as the control group.

After the women in both groups had signed informed consent forms, their basic clinical characteristics and obstetrics history (age, number of pregnancies, and gestational age) were gathered using a questionnaire. Body mass index (BMI) was calculated from weight and height and is expressed as weight in kg/height in square meters. Three milliliters of whole blood was collected from each participant in ethylene diamine tetra acetic acid‐coated tubes, and total DNA was extracted using the salting out method (Miller, Dykes, & Polesky, 1988).

### 2.3 SNP selection and genotyping

The rs3025039, rs16944, and rs1143634 polymorphisms were detected by examining the size of the polymerase chain reaction (PCR) products generated by DNA amplification of the *VEGFA* (NG_008732.1) and *IL1β* (NG_008851.1) target sequences. The *VEGFA* and *IL1β* genotypes were confirmed by sequencing the PCR products using the same oligonucleotides pairs (sequencing was performed by Macrogen Inc., South Korea). Details of the primers and enzymes used for PCR amplifications and the polymerase chain reaction–restriction fragment length polymorphism digestion results for the *VEGFA* and *IL1β* polymorphisms are shown in Table 1. All PCR reactions were performed in 25‐µl volumes comprised 13 µl ultrapure water, 5 µl reaction mix, 1 µl each of the forward and reverse primers, and 5 µl of purified DNA. The PCR conditions for detection of rs16944 were as follows: initial denaturation at 94°C for 5 min; 35 cycles of denaturation at 94°C for 30 s, annealing at 58°C for 30 s, and extension at 72°C for 30 s; and final extension at 72°C for 7 min. For rs1143634: initial denaturation at 95°C for 5 min; 35 cycles of denaturation at 94°C for 30 s, annealing at 62°C for 30 s, and extension at 72°C for 30 s; and final extension at 72°C for 7 min. For rs3025039: initial denaturation at 95°C for 5 min; 35 cycles of denaturation at 94°C for 45 s, annealing at 61°C for 45 s, and extension at 72°C for 45 s; and final extension at 72°C for 7 min. Then, each PCR product was digested using different restriction enzymes for each polymorphism: rs16944 (*AvaI,* Thermo Scientific), rs1143634 (*TaqI,* Thermo Scientific), and rs3025039 (*NlaIII,* Thermo Scientific) for 37°C for 24 hr in a 21‐µl reaction volume. Then, digested products were separated by gel electrophoresis on a 2.5% agarose gel and stained with ethidium bromide.

**Table 1:** Oligonucleotides, restriction enzymes used for detection of *VEGFA* and *IL1β* gene polymorphisms and genotyping result interpretation

Polymorphism	Primers pair	Restriction enzyme used	Fragments
*VEGFA* (rs3025039)	Forward 5′‐TGAAGGAGAAGGTGTCTGCGGGA‐3′ Reverse 5′‐AGGACGGTG CGGTGAGAGTG‐3′	*NlaIII*	(CC: 208 bp) (TT: 122 bp, 86 bp) (CT: 208bp, 122 bp, 86 bp)
*IL1β* (rs16944)	Forward 5′‐TGGCATTGATCTGGTTCATC‐3′ Reverse 5′‐GTTTAGGAATCTTCCCACTT‐3′	*AvaI*	(TT: 305 bp) (CC: 190 bp and 115 bp) (CT:305bp, 190 bp, and 115 bp)
*IL1β* (rs1143634)	Forward 5′‐GTTGTCATCAGACTTTGACC‐3′ Reverse 5′‐TTCAGTTCATATGGACCAGA‐3	*TaqI*	(TT: 250 bp) (CC: 138 bp and 112 bp) (CT: 250 bp, 138 bp, and 112 bp)

Note

*VEGFA* (NG_008732.1) and *IL1β* (NG_008851.1) gene.

The sample size was calculated based on a 1:1 ratio of cases to controls and the predicted difference in the rate of rs16944 and rs1143634 polymorphisms between the two groups. It was assumed that rs16944 and rs1143634 would be present in 55% of the pregnant women with preeclampsia and eclampsia and in 35% of pregnant women with no complication, based on a previous report (Leme Galvão et al., 2016). This would give the study at least 80% power to detect differences of 0.05 at the *α* level (Das, Mitra, & Mandal, 2016).

### 2.4 Statistics

The collected data were entered into SPSS for Windows for data analysis. The clinical data were compared between the two groups using *t* tests and chi‐square tests for continuous and categorical data, respectively. The observed and expected genotype distributions and their consistency with Hardy–Weinberg equilibrium (HWE) were assessed by Pearson's chi‐square (χ^2^) statistical test (Michael, 2008). Differences in allele frequencies between the cases and the controls were compared by Pearson's chi‐square statistical test. The risk associated with a specific genotype was estimated by univariate analysis and odds ratio and is expressed with a 95% confidence interval. A two‐sided *p* value < .05 was considered statistically significant.

## 3 RESULTS

There was no significant difference in the age, parity, BMI, or other characteristics tested between the women with preeclampsia and the healthy controls (60 women in each arm), as shown in Table 2.

**Table 2:** Comparing the mean (*SD*) of the sociodemographic characteristics between women with preeclampsia and the controls

Variables	Preeclampsia (60)	Controls (60)	*p‐*value
Age, year	27.7 (4.1)	27.9 (3.9)	.702
Parity	2.9 (1.7)	2.8 (1.6)	.643
Body mass index, Kg/m^2^	24.6 (1.9)	24.7 (2.1)	.444
Hemoglobin, g/dl	10.8 (1.5)	10.9 (1.7)	.339

The rs3025039, rs16944, and rs1143634 genotype distributions were consistent with the HWE (*p* > .05).

The rs3025039 polymorphism was significantly associated with preeclampsia (odds ratio [OR] = 2.49; 95% CI = 1.17–25.27; *p* = .01). The proportion of heterozygotes (CT) was significantly higher in the patient group: 45.0% compared with 26.7% of the control group, *p = *.02 (OR = 2.4, 95% CI = 1.12–5.32). The proportion of the T allele was significantly higher in women with preeclampsia compared with the healthy controls (29.2% vs. 26.7%, OR = 2.05, 95% CI = 1.10–3.83; *p* = .02), as shown in Table 3.

**Table 3:** Comparing the genotypes and alleles of *VEGFA* rs3025039 between women with preeclampsia and the controls

Genotypes	Preeclampsia (60)		Controls (60)		OR (95% CI)	*p*‐value
	*N*	%	*N*	%		
CC	29	48.3	42	70.0	Reference	
CT	27	45.0	16	26.7	2.4 (1.12–5.32)	.023
TT	4	6.7	2	3.3	2.89 (0.49–16.87)	.220
CT+TT	31	51.6	18	30.0	2.49 (1.17–25.27)	.015
Allele T	35	29.2	20	26.7	2.05 (1.10–3.83)	.021
Allele C	85	70.8	100	83.3	Reference	

Note

OR, odds ratio. *VEGFA* (NG_008732.1).

Heterozygosity at rs16944 was significantly associated with preeclampsia (OR = 2.55; 95% CI = 1.15–5.56; *p* = .01). However, there was no significant difference between the proportion of the C allele in women with preeclampsia (25.8%) compared with that observed in the healthy controls (20.0%) (OR = 1.39, 95% CI = 0.76–2.55; *p* = .28), as shown in Table 4.

**Table 4:** Comparing the genotypes and alleles of *IL1β* rs16944 between women with preeclampsia and the controls

Genotypes	Preeclampsia (60)		Controls (60)		OR (95% CI)	*p*‐value
	*N*	%	*N*	%		
TT	31	51.7	41	68.3	Reference	
TC	27	45.0	14	23.3	2.55 (1.15–5.56)	.019
CC	2	3.3	5	8.3	0.52 (0.09–2.91)	.458
TC+CC	29	48.3	19	31.6	2.01 (0.96–4.24)	.062
Allele C	31	25.8	24	20.0	1.39 (0.76–2.55)	.283
Allele T	89	74.2	96	80.0	Reference	

Note

OR, odds ratio. *IL1β* (NG_008851.1) gene.

rs1143634 was significantly associated with preeclampsia (OR = 4.50; 95% CI = 2.06–9.81; *p* < .001). The proportion of heterozygotes (CT) was significantly higher in the patient group (51.7%) compared with the control group (18.3%) (*p < *.001, OR = 5.28, 95% CI = [2.26–12.33]). Moreover, the proportion of women with preeclampsia carrying the T allele was significantly higher compared with the healthy control group (34.2% vs. 15.8%, OR = 2.75, 95% CI = 1.48–5.12, *p* = .001), as shown in Table 5.

**Table 5:** Comparing the genotypes and alleles of *IL1β* rs1143634 between women with preeclampsia and the controls

Genotypes	Preeclampsia (60)		Controls (60)		OR (95% CI)	*P*‐value
	*N*	%	*N*	%		
CC	24	40.0	45	75.0	Reference	
CT	31	51.7	11	18.3	5.28 (2.26–12.33)	<.001
TT	5	8.3	4	6.7	2.34 (0.57–9.55)	.225
CT+TT	36	60.0	15	25.0	4.50 (2.06–9.81)	<.001
Allele T	41	34.2	19	15.8	2.75 (1.48–5.12)	.001
Allele C	79	65.8	101	84.2	Reference	

Note

OR, odds ratio. *IL1β* (NG_008851.1) gene.

## 4 DISCUSSION

The major finding from the current study is the significant association between a *VEGFA* gene polymorphism and preeclampsia. Our finding is in line with Shim *et al* and Procopciuc *et al,* which reported significant associations between preeclampsia and rs3025039 (Procopciuc, Caracostea, Zaharie, & Stamatian, 2014; Shim et al., 2007). Interestingly, in their meta‐analysis including 11 studies (1,069 patients), Cheng *et al* (Cheng, Hao, Zhou, & Ma, 2013) showed that women with the rs3025039 T allele are at a 1.52 higher risk of developing preeclampsia (Cheng et al., 2013). Moreover, even though the T allele was the minor allele in our study (29.2%), it was significantly associated with preeclampsia; this finding is consistent with those reported by Shim *et al* in South Korea (26.8%) and by Papazoglou *et al* (20.2%) in Greece (Papazoglu et al., 2004; Shim et al., 2007). However, other studies have found no association between preeclampsia and rs3025039 (Andraweera et al., 2013b; Gannoun et al., 2017). It is worth mentioning that rs3025039 is located in the 3′‐untranslated region of *VEGFA* and can modulate *VEGFA* expression (Jain et al., 2009; Renner, Kotschan, Hoffmann, Obermayer‐Pietsch, & Pilger, 2000; Stevens, Soden, Brenchley, Ralph, & Ray, 2003). This activity is attributed to the presence of a binding sequence for hypoxia‐inducible factor‐1, which acts as a transcriptional factor for *VEGFA,* in the *VEGFA* 3′‐untranslated region (Levy, Levy, & Goldberg, 1996). Recently, Dong reported that carriers of the T allele have significantly higher levels of plasma VEGFA compared with those who carry the C allele (Dong, 2019).

The current study showed a significant association between rs16944 and preeclampsia. This finding is consistent with a single report from China (Wang et al., 2014). However, studies conducted in Taiwan, Holland, and the United States reported no association between rs16944 and preeclampsia (Hefler, Tempfer, & Gregg, 2001; Kang, Chen, Yu, Chang, & Chang, 2012; Lachmeijer et al., 2002). In this study, we found no association between the TT or CC genotype and preeclampsia; only the heterozygous TC genotype was significantly associated with preeclampsia. In the Chinese study, women with the CC genotype had a greater risk of developing preeclampsia, as well as for those harboring the T allele (Wang et al., 2014). In the current study, we identified a significant association between the rs1143634 polymorphism and preeclampsia. This polymorphism is located in exon number 5 of the coding region of the *IL1β* gene (Lachmeijer et al., 2002). We observed that women carrying the T allele were 2.75 times more likely to develop preeclampsia compared with those carrying the C allele. Moreover, women with CT+TT were 4.5 times more likely to develop preeclampsia. However, these results do not agree with any previous studies. Two studies from Iran failed to show any association between preeclampsia and rs1143634 (Tavakkol Afshari et al., 2016; Mohajertehran et al., 2012). Likewise, another study from Brazil found no association between preeclampsia and rs1143634 (Leme Galvão et al., 2016). This discrepancy could be attributable to ethnic differences between the study groups. Moreover, preeclampsia is a complex multifactorial disease, and interactions between environmental, immunological, infectious, lifestyle, and genetic factors can affect its development (Pennington, Schlitt, Jackson, Schulz, & Schust, 2012).

To the best of our knowledge, this is the first study to investigate genetic factors associated with susceptibility to preeclampsia in Sudan specifically and sub‐Saharan Africa more generally. Identifying patients at risk of developing preeclampsia is of paramount importance for healthcare providers. In this study, we detected an association between preeclampsia and *VEGFA* and *IL1β* gene polymorphisms. However, the study had some limitations that should be addressed in future studies. First, we did not quantify plasma VEGFA and IL‐1β levels in the studied population. This information would be useful for confirming the functional effects of these polymorphisms and could help explain the clinical presentation of the patients. Second, we investigate selected SNPs in just two genes based on a candidate gene approach. Investigating haplotypes rather than SNPs could be a more useful approach for future studies. Third, although our sample size was calculated to provide 80% power, a larger sample is needed to ensure the precision of the results. Thus, further study is needed.

## CONFLICT OF INTEREST

All authors declare that they have no competing interests.

## AUTHORS' CONTRIBUTIONS

HMH, SEA, and IEA carried out the study and participated in the statistical analysis and procedures. MS and HMH carried out the study. HZH and IA helped design the study, performed the statistical analysis, and drafted the manuscript. All the authors read and approved the final version of the manuscript.
